# Pulmonary Pathogen-Induced Epigenetic Modifications

**DOI:** 10.3390/epigenomes7030013

**Published:** 2023-07-06

**Authors:** Dylan Wrede, Mika Bordak, Yeabtsega Abraham, Masfique Mehedi

**Affiliations:** School of Medicine & Health Sciences, University of North Dakota, Grand Forks, ND 58202, USA; dylan.wrede@und.edu (D.W.); mika.bordak@und.edu (M.B.); yeabtsega.abraham@und.edu (Y.A.)

**Keywords:** virus, epigenetics, bacteria, fungi, histone modification, DNA methylation, RSV, transcriptional factors

## Abstract

Epigenetics generally involves genetic control by factors other than our own DNA sequence. Recent research has focused on delineating the mechanisms of two major epigenetic phenomena: DNA methylation and histone modification. As epigenetics involves many cellular processes, it is no surprise that it can also influence disease-associated gene expression. A direct link between respiratory infections, host cell epigenetic regulations, and chronic lung diseases is still unknown. Recent studies have revealed bacterium- or virus-induced epigenetic changes in the host cells. In this review, we focused on respiratory pathogens (viruses, bacteria, and fungi) induced epigenetic modulations (DNA methylation and histone modification) that may contribute to lung disease pathophysiology by promoting host defense or allowing pathogen persistence.

## 1. Introduction

Pulmonary pathogens have been responsible for some of the biggest global health crises in humans for centuries. This is in part due to the efficiency of their mode of transmission. However, their ability to manipulate the human immune system has also been observed as a significant factor. It has already been known that epigenetic alterations play an important role in pathology as well as their ability to modify the immune response [1]. Epigenetic modifications associated with nutrition and the microbiome and their contribution to long-term health development have been well documented [2]. Importantly, the recent research successes show how pulmonary pathogens can modulate the human genome and cause chronic diseases due to the advancement in the OMICS sciences, such as genomics, transcriptomics, proteomics, or metabolomics. For example, genome-wide association studies have revealed genetic changes in different regions of the genome that are primarily associated with complex and nonmalignant respiratory diseases, e.g., chronic obstructive pulmonary disease (COPD), asthma, and pulmonary arterial hypertension (PAH) [3,4,5]. Benincasa et al., have reviewed the epigenetic role in COPD, asthma, and PAH [6]. The epigenetic markers for these common chronic respiratory diseases were identified, e.g., DNA methylation, histone modification, and microRNA (miRNA) [7,8,9,10]. There is growing evidence supporting bacterial infection-induced epigenetic modification in the host by different mechanisms [11]. Viruses are intracellular parasites that continuously utilize the subordination and exploitation of cellular machinery for transcription and translation. Thus, it is no surprise that viruses induce modulation of host chromatin dynamics and transcription regulation, and examples of modulated epigenetic mechanisms are DNA methylation, histone post-translational modification, and transcription modification [12,13]. Bacterial virulence factors can modulate host cell genetic expression rates, limiting the ability of the immune system to adequately respond and allowing replicating bacteria to evade phagocytosis. Multiple virulence factors have been identified in recent studies that may be able to cause lasting pathogenic changes to host cell genomes, some of which may be associated with chronic pathologies such as allergy development, asthma, latent infection, carcinogenesis, and COPD exacerbation. Previously described epigenetic genome modifications, including histone modification, miRNA processing, DNA methylation and acetylation, protein chaperones, and protein degradation processes, all have the potential to upregulate and downregulate host genes at the whim of the invading organism [1,14,15]. These epigenetic changes have potentially chronic or permanent consequences for the host, which can last long after the resolution of the initial infection due to the persistence of some of these changes throughout generations of proliferating cells. Linking the virulence factors of common respiratory infections to consistent host genome modifications and finally to measurable long-term pathogenic outcomes is a potential new front for pulmonary research. The field of infectious epigenetics is still in its early stages, but many authors have already extensively cataloged the most common pathogens and their respective epigenetic targets [1,14,15]. 

In this review, we have discussed pulmonary pathogens (viruses, bacteria, and fungi) induced epigenetic alterations and their role in typical cell pathology. We highlighted the current understanding of how host-bacterial, viral, or fungal infections contribute changes to the epigenetic landscape and whether those interactions are responsible for the downstream effects contributing to chronic pulmonary diseases. 

## 2. Epigenetic Mechanisms Regulate Gene Expression

**Figure 1:**
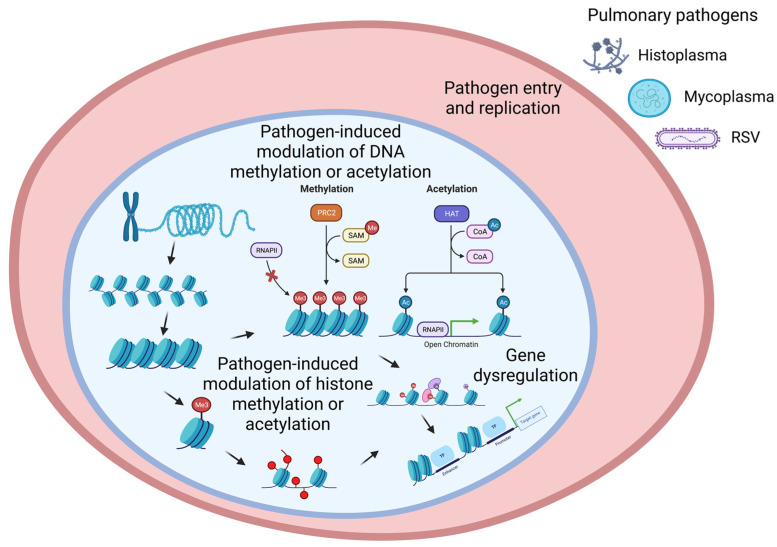
A schematic of pulmonary pathogen-induced modulation of epigenetic factors. Pulmonary pathogens: bacterium (e.g., mycoplasma), virus (e.g., RSV), and fungus (e.g., histoplasma). Two major pathogen-driven epigenetic modulations of gene regulation: 1. DNA methylation or acetylation, and 2. Histone methylation or acetylation.

The cells of eukaryotes are genetically homogenous but phenotypically heterogenous due to the differential expression of genes. One of the ways of regulating differential gene expression is epigenetically, which does not need mutation but is heritable [16]. Pathogens (bacterium, virus, and fungus) are known to modulate diverse epigenetic factors, namely histone modification, DNA methylation, chromatin-associated complexes, non-coding RNAs, and RNA splicing factors [11]. Here, we highlighted two major epigenetic mechanisms of gene regulation that have been reported to be modulated by pathogens (Figure 1): histone modifications [17] and DNA methylation [18].

### 2.1. Histone Modification

Histone modification is the host cell’s ability to condense transcriptionally active euchromatin into highly condensed and silenced heterochromatin. The 147 base pairs of DNA wrapped around the histone octamers create a superstructure called a nucleosome that provides a highly ordered scaffold for condensing and storing DNA, and also uses steric hindrance to restrict the access of RNA polymerase enzymes to the incorporated DNA strand and any included genes [17]. The host cell can use writer proteins to covalently modify the N-terminal histone tails at more than 200 different sites with phosphorylation, acetylation, acylation, citrullination, methylation, or ubiquitination to change their properties of interaction with surrounding nucleosomes. The specific pattern of histone modification can either move nucleosome complexes along the strand of DNA, exposing particular genes for transcription, or create transcriptionally silent supercoils [19]. Each post-translational modification exerts electrochemical “cross-talk” on other covalent groups on the histone, creating a complex pattern of modification that can drastically influence the way a cell behaves. Proteins called erasers are responsible for removing these post-translational modifications, creating a dynamic balance between writing and erasing these groups that can be quickly modulated to respond to an infectious threat [20]. Multiple species of bacteria can secrete virulence factors that can directly remove the covalent modifications on the histone tails, modulating transcriptional activity for their own benefit [14,21]. Many virulence factors have the effect of slowing the immune response by suppressing transcription of proinflammatory proteins, thereby inhibiting immune cascades and the recruitment of additional immune cells [17]. *Chlamydia trachomatis* uses this mechanism by secreting a nuclear effector protein with histone N-lysine methyltransferase activity and histone demethylase activity to specifically modulate the methylation of histones H2B, H3, and H4. This subversion of host transcription can cause a global reduction in overall genetic activity and severely limit the cell’s ability to secrete alarm chemokines [22].

Another pathogenic histone modification mechanism can be accomplished through the modulation of histone-modifying enzymes, impairing the host’s ability to sense and respond to immune threats. Acetylation of histone tails at lysine residues creates a net negative charge, which can repel nearby nucleosomes and cause the chromatin to uncoil, but can have other varying effects based on other local post-translational modifications and their intrinsic cross-talk [23]. HDAC (Histone Deacetylase) proteins are responsible for removing acetyl groups when necessary, which often creates a heterochromatin state and silences genes [23]. This process has been shown to be upregulated during infections with *Anaplasma phagocytophilum*, *Mycobacterium tuberculosis,* and *Pseudomonas aeruginosa*, limiting the host cell’s ability to transcribe genes and respond to the infection. A well-defined example is the development of bacterial virulence factors that mimic the Ankyrin A protein and directly recruit and activate the HDAC1 enzymes at the site of transcriptionally active euchromatin. This causes localized hypoacetylation and gene suppression at the CYBB gene, which is essential for superoxide anion production and the proper function of the NADPH oxidase reaction [24].

### 2.2. DNA Methylation

DNA methylation is an easy way for a host cell to functionally silence transcription of a gene without excising the genetic code completely. DNA methyltransferases can quickly add a methyl group to a gene of interest at a cytosine or adenosine residue, creating methyl-cytosine or methyl-adenosine [18]. These bulky chemical groups inhibit the attachment of transcription factors and downregulate the activity of RNA polymerase and subsequent gene expression. Methyl groups will virtually silence all transcription, and acetyl groups tend to increase RNA polymerase activity at the site in question. These changes are persistent throughout generations of progenitor cells, creating chronic consequences for the host. Recognizing deleterious genomic methylations in a host cell that were specifically caused by an infection is difficult, and because of this, few pathogens have been associated with epigenetic methylations. Host cell methylations are usually localized to CpG islands in the genome, areas rich in cytosine and guanine residues, which allow for the activity of DNA-methyltransferase. Non-CpG methylations are one of the few true markers of pathogenic methylation [25]. Perhaps one of the best-characterized examples of this effect has been demonstrated by *Mycobacterium tuberculosis* Rv2699 protein secretion within host macrophages [25,26]. Multiple studies have shown various de novo methylations associated with Rv2699 proteins in macrophages infected with *M. tuberculosis*, with a possible decrease in IL-6 expression in infected cells [14]. Current reviews have also highlighted many different temporary epigenetic mechanisms used by pathogens to enhance virulence [1,14]. These changes are not passed through host progenitor cell lineages, so they are unlikely to cause chronic disease after infection, but many of these mechanisms may lead to clinically significant effects on the host beyond what may have been directly caused by the pathogen. Many questions remain about the long-term potential consequences of these infections. The exact mechanisms for the oncogenic epigenetic impacts of some infections like *Helicobacter pylori* and *Human papillomavirus* have been extensively documented [27,28,29,30].

## 3. Bacterial Infection-Mediated Pulmonary Epigenetics

Bacterial infections causing epigenetic change within a host nasal epithelial cell genome have been newly credited for being a significant pathogenic contributor to future chronic disease [11,31]. Ebenezer et al., have shown *Pseudomonous aeruginosa* infection drives epigenetic regulation in the host. They used the *P. aeruginosa* mouse model to test how the infection regulates nuclear spingosine-1-phosphate kinase 2 (SPHK2) and spingosine-1-phosphaet (SP1) and modulates epigenetic regulation in ling injury in vivo [32]. An in vitro study has also shown pathogenic bacterial infection-induced epigenetic modulation, e.g., flagellin of *Legionella pneumophila* is known to modulate histone (H3) acetylation or phosphorylation in infected lung epithelial cells [11]. Importantly, respiratory tract commensal bacteria, e.g., *Moraxella catarrhalis*, were also found to modulate acetylation or phosphorylation of H3 via the induction of an inflammatory signaling cascade (Figure 1) [33,34]. Denzer et al. [14] and Rajeev et al. [1] have comprehensively reviewed current research on epigenetic changes caused by bacterial infections, including many demonstrated epigenetic mechanisms of modification. Dupont et al. [35] define epigenetics as a change in gene function that is mitotically and/or meiotically heritable and that does not entail a change in DNA sequence. This is a rather classical perspective and excludes extra-genomic modifications done to the cell that have drastic effects on phenotype yet are not passed through progenitors. Interestingly, intrinsic, acquired, or adaptive forms of antimicrobial resistance can be characterized by different genetic and epigenetic molecular pathways [36,37,38]. Many current reviews of epigenetics have now expanded that definition to include those changes that are not passed through DNA replication and instead only affect cells directly modified by genetically active virulence factors [39]. For the purposes of this review and studying the effect of these diseases on the development of chronic ailments, we focused on the heritable epigenetic changes shown by human epithelial cells after a bacterial infection (Table 1).

## 4. Viral Infection-Mediated Pulmonary Epigenetics

Respiratory viral infections can trigger chronic lung diseases [63,64,65]. Asthma is a common chronic disease affecting children in Western countries. Although the pathophysiological changes underlying asthma development are complex and unknown, respiratory syncytial virus (RSV) and human rhinovirus virus (HRV) have been recognized as major etiological factors in wheezing illness, with a significant correlation with asthma development during childhood [66,67]. A recent number of studies have shown that respiratory viral infection causes epigenetic changes, which refer to genetic alterations that affect gene expression without any mutational genetic changes [64]. Here, we focused on a few common respiratory virus-driven epigenetic changes (Table 2 and Table 3). 

### 4.1. Adenovirus

Adenovirus is a double-stranded DNA virus that commonly causes respiratory infections in humans. It uses a lytic type of life cycle, which is important in its pathophysiology [68]. A common theme in adenovirus infection is the suppression of the expression of most host genes except a few that are involved in cell division [69]. These profound changes are believed to be caused in part by epigenetic changes. ChIP-sip and ChIP-seq-based data analyses suggested that Adenovirus infection was associated with the modulation of two major epigenetic regulations: the organization of certain histone modifications and transcription factors within infected cells. The adenovirus e1a protein appeared to affect the acetylation of H3K9 and H3K18 and certain transcriptional factors [70,71]. Adenovirus protein VII is expressed late in the infected cells and translocates into the nucleus, which allows it to associate with host chromatin; consequently, it alters the chromatin composition of the infected cells. [72,73]. Additionally, the Adenovirus e1A protein is known to localize to host chromatin, alter host histone modifications, and overhaul transcription in infected cells. e1A binds to three different locations on the host’s chromatin. The first site is a region occupied by multiple genes related to the immune response of the host, and e1A binding downregulates these genes [72]. The second site of binding for this protein involves genes involved in the cell cycle. These genes were observed to have been upregulated [68,72]. The genes at the third site of binding are mostly involved in specialization and growth, and these genes were found to have been downregulated. These epigenetic changes hinder the immune system’s ability to respond to an adenovirus infection [72].

### 4.2. RSV

RSV is a negative-sense, single-stranded RNA virus enveloped by a protein coat. Since RSV affects the epithelial cells of the respiratory tract, the epigenetic factors that may induce changes in these cells are of particular interest. Caixia et al., underlined how the epigenetic dysregulation of signaling pathways in epithelial cells of the respiratory tract is related to shortfalls in immunological functions [74]. This implies that, through the induction of epigenetic modification, the virus can offset the immune response against it. Some of the signaling pathways that RSV can impact are tyrosine kinase growth factor signaling, the hexosamine biosynthetic pathway, and the extracellular matrix secretory pathways [75]. The virus can do this by inducing chromatin remodeling, which involves an increase in the number of nucleosome-free regions. This remodeling affects genes that regulate the aforementioned pathways. The increased accessibility of specific chromatin regions directly affects TGFβ-ECM and HBP pathways, which may explain the airway remodeling that happens in RSV infection [75].

Fonseca et al. [76] discussed that the disparities in immune responses between adults and infants are caused by epigenetic alterations of inflammatory genes. Histone methylation caused by RSV infection has been seen to enhance the production of Th2 cytokines after diminishing the production of pro-inflammatory cytokines. Other observations that have been made in mouse models suggest that RSV is also able to induce changes in non-coding RNAs [76]. These changes were seen to have impacts after the infection had subsided, which involved allergic asthma [76]. By having these effects, the virus is able to increase its own virulence while also having lasting effects, especially on vulnerable hosts.

### 4.3. Severe Acute Respiratory Syndrome Coronavirus 2 (SARS-CoV-2)

SARS-CoV-2 is a positive-sense, single-stranded RNA virus that was first identified in the city of Wuhan, China. It is the virus responsible for the respiratory disease coronavirus disease 2019 (COVID-19). The virus has been found to alter the epigenetic environment of the host cell to increase its virulence by disrupting the host’s ability to recognize and respond to the pathogen [77]. Arguably the most significant regulator of the severity of COVID-19 infection is correlated with the increased expression of the receptor ACE2. DNA methylation is one way in which this receptor can be altered by the virus. Decreased DNA methylation close to the transcription site was found to result in increased expression of ACE2 in lung epithelial cells [77]. Chlamydas et al., suggested that the upregulation of the ACE2 receptor on the host cells by the virus is through histone modifications at the DNA packaging histones H3, H3K4me1, H3K4me3, and H3K27Ac [77]. Ozturkler et al., summarized how epigenetic changes such as hypermethylation of IFN genes and hypomethylation of inflammatory genes can be induced by the virus, and this change can be cell-specific [78]. Corley et al., described how, in severe cases of COVID-19, the epigenetic changes in immune cells varied and DNA methylation decreased in primary neutrophils [79].

**Table 2:** Common viral pulmonary pathogens and their associated epigenetic changes.

Pulmonary Pathogen	Epigenetic Changes	Cellular Impact	References
Adenovirus	Histone deacetylase inhibitor suppresses host HDAC proteins	Increased global acetylation, transcription modulation	[68,72]
Respiratory syncytial virus (RSV)	Chromatin modifications inducing nucleosome-free regions Demethylation of Nodal promoter Increased TGFβ expression	Impacts tyrosine kinase growth factor signaling, affects extracellular matrix secretory pathways Reduced pro-inflammatory cytokines release	[74,75,76]
Severe acute respiratory syndrome coronavirus 2 (SARS-CoV2)	Hypermethylation of IFN related Hypomethylation of inflammatory genes Perturbation of epigenetic clock IRF1 and IRF7 were upregulated Epi-factors HDAC9 and SIRT1 were deregulated.	Increased level of ACE2 receptor expression Increased cytokine release	[77,78,79,80]
Influenza virus	Post translational histone modification Decrease in histone acetylation Demethylation on CREB1 binding region	Hypercytokinemia	[81,82,83]
Rhino virus	Modifications in DNA methylation		[64]
Middle East respiratory syndrome-related corona virus (MERS)	H3K27 methylation	Downregulate antigen-presenting molecules	[13,77,83]

TGFβ: tumor growth factor β; IFN: interferon; IFR1: interferon regulatory factor 1; IRF7: interferon regulatory factor 7; SIRT1: sirtuin 1; ACE2: angiotensin converting enzyme 2; CREB1: CAMP responsive element binding protein 1; H3K27: histone 3 at lysine residue in position 27.

**Table 3:** Main types of epigenetic changes and virus presence in common respiratory viral diseases.

Types of Epigenetic Change	Adenovirus	SARS-CoV-2	RSV	Influenza Virus	Rhino Virus	MERS
Chromatin remodeling	Not Identified	Not Identified	Yes [75]	Yes [83]	Not Identified	Not Identified
Changes in DNA methylation	Yes [68]	Yes [77,78,79]	Yes [74]	Yes [81,82]	Yes [64]	Yes [83]
Non-coding RNA		Yes [80]	Yes [74]			
Changes in DNA acetylation	Yes [68]	Not Identified	Not Identified	Yes [81]	Not Identified	Not Identified

## 5. Fungal Epigenetics

Human pathogenic fungi-induced epigenetic changes are not well studied like viruses and bacteria, although there is a plethora of research into plant pathogenic fungi [84]. One cause for this lack of human-centered research could be the population affected by pathogenic fungi. Most fungal infections, including fungal respiratory infections, are asymptomatic in healthy populations [85]. Patients with asymptomatic fungal infections are less likely to seek medical care and thus pose no additional burden on the healthcare system. However, when pathogenic fungi infect vulnerable or immunocompromised patients, the infections may become invasive and require hospitalization [86,87,88]. A smaller patient population that is afflicted with fungal infections may be less enticing to researchers as compared with the vast population that can be infected with a bacterium like *Streptococcus pneumoniae* [88,89].

However, despite the smaller proportion of patients affected by fungal disease, the burden of fungal infections on the United States healthcare system should not be understated. A recent study from the Open Forum on Infectious Diseases, a publication from the Infectious Diseases Society of America, shows that fungal infections cost the United States approximately $6.7 billion in 2018. This study attributes 76.3% of the fungal infections and 81.1% of the associated costs to three pathogens: *Aspergillus*, *Pneumocystis*, and *Candida* [90]. All three of those fungi cause severe pulmonary disease in immunocompromised patients. 

Fungal infections can impact the treatment and care of patients who are both immunocompromised and immunocompetent. In patients with chronic conditions like COPD or asthma, additional fungal infections complicate their care and may cause a severe exacerbation that requires hospitalization. A key factor contributing to all three chronic diseases (asthma, COPD, and cystic fibrosis) is mucus hypersecretion. Although epigenetic regulation of mucus hypersecretion remains unknown, a comprehensive review of epigenetic research on the mucus hypersecretion aspect of these chronic diseases reveals genes associated with DNA methylation and histone modification are major contributing factors [91]. One indication of fungal infection-induced epigenetic changes could be extrapolated from the funding by Perez FJ et al. They showed that fungal colonization with *Pneumocystis* has been shown to elevate the concentration of chloride channel accessory 1 (hCLCA1) in the infant lung parenchyma, and it also correlates with overproduction of mucin 5 AC (MUC5AC) [92]. Co-infections of fungal disease and viral infections, such as a co-infection of *Aspergillus* and SARS-CoV-2, can complicate the treatment of both diseases and have been attributed to poorer patient outcomes [93]. 

## 6. Conclusions

Genome-wide studies for characterization of epigenetic changes during pulmonary pathogen infection provide a better understanding of the complex relationship between pathogen infections and pulmonary diseases and even chronic lung diseases, e.g., asthma and COPD. However, we need to emphasize whether epigenetic changes directly correlate with changes in biological functions. Less common pathogens have still not been fully studied in the context of epigenetic modulations, and notably, neither have the long-term clinical consequences of these infections. Identifying which pathogens pose the greatest risk for the development of chronic disease and delineating the mechanism of their epigenetic changes can help focus public health efforts on the prevention or elimination of those diseases.

## Figures and Tables

**Table 1:** Common pulmonary diseases and their associated epigenetic impacts due to bacterial infections.

Pulmonary Pathogen	Epigenetic Changes	Cellular Impact	References
*Actinomyces* spp.	Histone deacetylase inhibitor suppresses host HDAC proteins	Increased global acetylation, transcription modulation	[1,40]
*Bordetella bronchiseptica*	bbSET17 methylates histones associated with rRNA genes	rRNA transcription modulation	[1,41]
*Burkholderia* spp.	btSET methylates H3	Methylation of H3K4 at rDNA promoter regions	[1,41]
	Suppression of HDAC1/2 activity	Histone hyperacetylation, suppression of DNMT3B	[1,42]
*Mycobacterium tuberculosis*	Activation of HDAC1, global deacetylation of H3	Suppresses IL-12B, IFN-Y	[14,43]
	Rv1988 methylates H3R42	Suppresses innate immunity genes	[1,44]
	Rv2416c acetylates H3 at IL-10 promoter.	Inhibits Th-1, host immune attenuation, protects m. tuberculosis from autophagy	[1,45]
	Rv2699c activates DNA methyltransferase activity at cytosine residues and histones H3, H4	Non-CpG methylation, global transcription suppression	[1,25]
	Rv3423.1 activates histone acetyltransferase	Activation of host anti-inflammatory cascades, protects infection	[1,46]
*Streptococcus pneumoniae*	Pneumolysin dephosphorylates H3S10	May impair cell proliferation, tumor suppression	[1,14,47]
	Pneumolysin activates miRNA-200b	Blocks KALRN, enhances pneumonia pathology	[1,48]
*Legionella pneumophila*	LegAS4 methylates H3K4	Increased transcription of rRNA/ribosomal protein	[1,14,49]
	RomA globally methylates H3K14	Global transcription suppression	[1,14,50]
	Snpl targets DSIF complex	Inhibits RNA polymerase II	[1,51]
	AnkH targets LARP7 (snRNP)	Inhibits transcription elongation and splicing	[1,52]
*Bacillus anthracis*	BaSET directly trimethylates the NFkB gene at H1 lysine	Shuts down NFkB proinflammatory cascade	[1,14,53]
	LT targets IL-8 promoter	Condensation of chromatin at H3S10ph and H3K14ac	[1,54]
	LT targets IL-1B enhancer region (HDAC8)	Deacetylation of H3K27ac	[1,55]
*Burkholderia thaliadensis*	BtSET methylates H3K4 at NFkB gene	Suppresses NFkB cascade; activates rRNA transcription	[14,41,53]
*Chlamydia trachomatis*	NUE methylation of H2B, H3, H4	Global transcription suppression	[1,14,22]
*Chlamydia Pneumoniae*	cpnSET methylates H3	Global transcription modulation/suppression	[1,56]
*Chlamydia psittaci*	SINC targets MAN1, LAMP1	Modulates chromatin anchoring on inner nuclear membrane.	[1,57]
	Excess methylation on CpG islands, CDH1 gene	Inactivation of E-cadherin expression	[1,58]
*Escherichia coli*	Membrane vesicles target H3 (methylation)	Increase in transcription of H3K4me3 genes	[1,59]
*Moraxella catarrhalis*	Phosphorylation of H3S10, acetylation of H3K14 at IL-8 gene	Induces inflammatory response, MAPK, NFkB activation, release of IL-8	[1,34]
*Pseudomonas* *aeruginosa*	2-amnoacetophone induces HDAC1 activity	H3K18 deacetylation, heterochromatin state, reduced expression of cytokines/chemokines	[1,14,60]
	2-amnoacetophone indirectly dephosphorylates H3	Global dephosphorylation of H3S10, transcription modulation	[1,61]
	miRNA-93 targets IL-8 transcripts	Suppression of IL-8 response	[1,61]
Neisseria *gonorrhea*	Gc-HDAC targets HDAC1, deacetylates at H3/Mir-146a	Condensation of chromatin at H2K9ac/suppression of immune response	[1,62]

bbSET17: *B. bronchiseptica* SET domain protein; btSET: *B. thailandensis* type III effector; DN;MT3: DNA methyltransferase 3; IL-10: interleukin 10; IL-12β: interleukin; IFN-γ: interferon gamma; Th-1T-helper 1; KALRN: Kalirin RhoGEF kinase; LegAS4: *L. pneumophila* type IV secrection system effector; DSIF: DRB sensitivity-inducing factor; AnkH: AnkH inorganic pyrophosphate transport regulator; LARP7: La-related protein 7; baSET: *B. anthracis* SET protein; NFκB: nuclear factor kappa B; LT: lethal toxin; IL-8: interleukin 8; IL-1β: interleukin 1 beta; NUE: nitrogen use efficiency; cpnSET: *C. pneumoniae* SET domain protein; MAN1: LEM domain-containing protein 3; LAMP1: lysosomal associated membrane protein 1; CDH1: cadherin 1; MAPK: mitogen-activated protein kinase.

## Data Availability

Not applicable.
